# Orthodontic retreatment: positive effects on the patient’s self-esteem and quality of life

**DOI:** 10.1590/2177-6709.26.5.e21bbo5

**Published:** 2021-10-25

**Authors:** Laíze Rosa Pires FREITAS, Dauro Douglas OLIVEIRA

**Affiliations:** 1 Pontifícia Universidade Católica de Minas Gerais, (Belo Horizonte/MG, Brazil).; 2 Private practice (Belo Horizonte/MG, Brazil).

**Keywords:** Quality of life, Oral health, Orthodontic retreatment

## Abstract

**Introduction:**

An increasing percentage of the world’s population has had access to orthodontic treatment within the last few decades. Consequently, a larger number of patients seeking for correction of their malocclusions, nowadays, present with a history of previous orthodontic therapy. Orthodontists performing retreatments in their practice may have to face additional difficulties, and one of them is treating individuals that may be even more demanding for excellent results and efficient treatments.

**Objectives:**

This manuscript discusses the challenges faced when performing orthodontic retreatments. It illustrates a two-phase retreatment of a pre-adolescent and the ortho-surgical retreatment of a young adult with high demands for fast and exceptional results. Finally, this paper elaborates on the positive impacts that these retreatments had on the patients’ self-esteem and quality of life.

## RESUMO

**Introdução:**

Um percentual crescente da população mundial vem tendo acesso ao tratamento ortodôntico ao longo das últimas décadas. Consequentemente, hoje em dia, um maior número de pacientes em busca da correção de suas más oclusões apresenta um histórico de tratamento ortodôntico prévio. Ortodontistas que realizam retratamentos ortodônticos podem enfrentar dificuldades adicionais, e uma delas é ter que lidar com indivíduos que apresentam demandas e expectativas ainda maiores em relação à obtenção de resultados excelentes e de forma bastante eficiente.

**Objetivos:**

O presente artigo discute os desafios e os impactos positivos do retratamento ortodôntico causados na autoestima e na qualidade de vida dos pacientes, e ilustra o retratamento em duas fases de uma pré-adolescente, e o retratamento ortodôntico-cirúrgico de uma paciente adulta jovem que tinha demandas muito elevadas para a obtenção de resultados rápidos e excelentes.

## INTRODUCTION

Orthodontic treatment has increasingly become available to a larger percentage of the population within the last decades.^1^ ^,^ ^2^ This is due to increased access to oral health care based on the preventive philosophies of contemporary Dentistry, greater longevity of the population, better access to information, higher aesthetic demands of modern society, technological advances in Orthodontics,^3^ ^-^ ^6^ and lower treatment costs in some countries. 

The positive effects of orthodontic treatment on patient’s quality of life have been clearly demonstrated.^7^ ^,^ ^8^ Children, adolescents and adults present a better body image and self-confidence related to their appearance after the completion of their orthodontic therapy, thus presenting lower levels of anxiety in social relationships and positive impacts on their self-esteem.^9^ ^,^ ^10^

An increasing proportion of the individuals pursuing orthodontic treatment nowadays present with a history of previous orthodontic therapy.^11^ The reasons why patients seek orthodontic retreatment are multifactorial, and may include: desire to further improve esthetics and oral function, inadequate retention phase, maturational changes, unfavorable skeletal growth, failure in diagnosis and treatment planning or poor treatment.^12^ ^,^ ^13^

Orthodontists performing retreatments in their daily practice might have to manage patients presenting higher demands in relation to treatment quality and duration. Therefore, for an orthodontist to retreat successfully, a strong understanding of the difficulties that could lead to failure is necessary. Furthermore, an objective system to guide the excellence in finishing retreatments could facilitate the clinical management of these cases and increase the chances of success.^4^ ^,^ ^14^

The purpose of this manuscript is to illustrate how the application of the objective criteria of the Brazilian Board of Orthodontics (BBO) assisted in achieving excellent finishing in two orthodontic retreatment patients.

### CASE 1

#### DIAGNOSIS

The parents of a 9.8-year-old female patient sought for a second opinion about the results of an interceptive orthodontic treatment performed in their daughter. According to the parents, the patient had been wearing a removable palatal crib to try to eliminate a thumb-sucking and tongue thrust habits, and to correct an open-bite without success. The patient mentioned that she had been bullied at school due to her unpleasant smile, and her parents reinforced how her self-esteem was low because she did not show her teeth upon smiling. Both her medical and dental history were within normal limits. 

**Figure 1:**
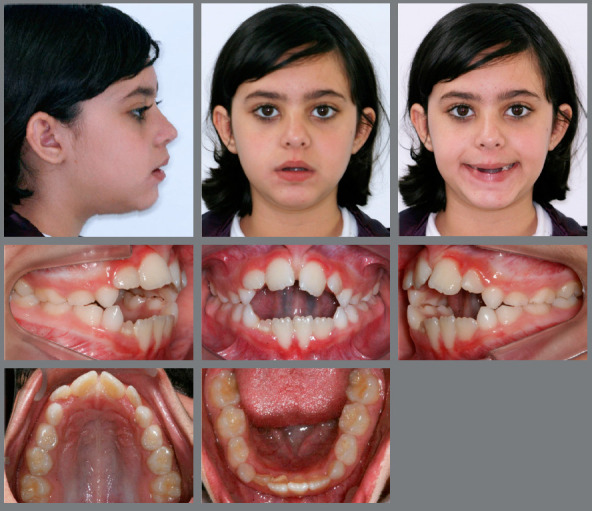
Initial facial and intraoral photographs.

Extraoral evaluation revealed a symmetric face, absence of passive lip sealing, moderately hyperdivergent growth pattern with an increased lower facial height and a slightly convex facial profile. Furthermore, the patient presented a significantly compromised smile esthetics without any maxillary incisors display upon smiling (Fig 1).

Intraoral analysis showed an Angle Class I malocclusion combined with a severe anterior open bite. Although no posterior crossbite was observed, there was a transverse maxillary deficiency, a tapered maxillary arch form and an increased palate depth. Mandibular posterior teeth presented increased lingual inclination as a compensation for the decreased maxillary transverse dimension. Mild crowding on both dental arches was also registered. Periodontally, there was a significant gingival recession on both mandibular central incisors, presence of calculus on the lingual surface of the lower incisors, and a low insertion of the upper lip frenum (Fig 1). 

**Figure 2:**
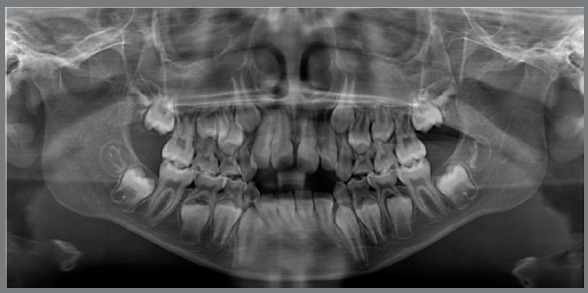
Initial panoramic radiograph.

**Figure 3:**
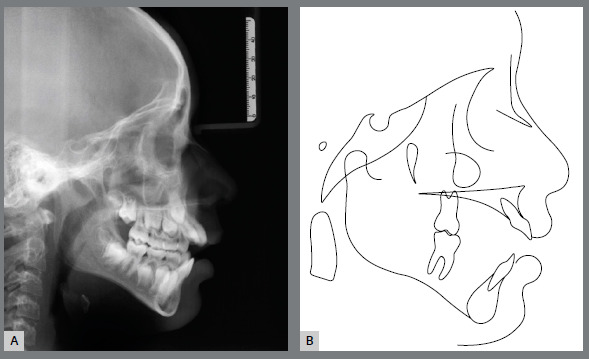
Initial cephalometric profile radiograph (A) and cephalometric tracing (B).

Panoramic radiograph confirmed the presence of all permanent teeth and a reduced eruption of all maxillary and mandibular incisors (Fig 2). Cephalometric evaluation revealed a Class II skeletal malocclusion (ANB = 7º; Witts = +6 mm), with a properly positioned maxilla (SNA = 82º), a retruded mandible (SNB = 75º) and a convex profile (Angle of convexity = 13º). There was an increased vertical dimension of the face (FMA = 30º SN.GoGn = 39º). Both maxillary and mandibular incisors presented normal bodily position (1-NA = 5 mm; 1-NB = 6 mm, respectively), but were significantly proclined (1.NA = 32º and IMPA = 97º, respectively) (Fig 3 and Table 1).

**Table 1:** Cephalometric values at start (A) and at the end (B) of treatment.

	MEASURES		Normal	A	B	A/B
Skeletal pattern	SNA	(Steiner)	82°	82°	82°	0
	SNB	(Steiner)	80°	75°	78°	3
	ANB	(Steiner)	2°	7°	4°	3
	Wits	(Jacobson)	♀ 0 ± 2 mm ♂ 1 ± 2 mm	6 mm	0 mm	6
	Angle of convexity	(Downs)	0°	13°	9°	4
	Facial Angle	(Downs)	87°	83°	88°	5
	SN.GoGn	(Steiner)	32°	39°	40°	1
	FMA	(Tweed)	25°	30°	30°	0
Dental pattern	IMPA	(Tweed)	90°	97°	98°	1
	1.NA (degrees)	(Steiner)	22°	32°	27°	5
	1-NA (mm)	(Steiner)	4 mm	5 mm	4 mm	1
	1.NB (degrees)	(Steiner)	25°	35°	37°	2
	1-NB (mm)	(Steiner)	4 mm	6 mm	8 mm	2
	- Interincisal angle	(Downs)	130°	111°	110°	1
Profile	Upper lip - S-line	(Steiner)	0 mm	0 mm	-1.8 mm	1.8
	Lower lip - S-line	(Steiner)	0 mm	0 mm	0 mm	0

#### TREATMENT PLANNING AND ORTHODONTIC MECHANICS USED

The previous interceptive orthodontic treatment achieved minor or no results with the use of a removable crib to interrupt the patient’s thumb sucking habit, due to the lack of cooperation reported by both parents and the patient herself. Additionally, the removable plate did not address the maxillary transverse deficiency that resulted from the unbalanced muscular pressures observed in patients presenting these types of deleterious oral habits. 

**Figure 4:**
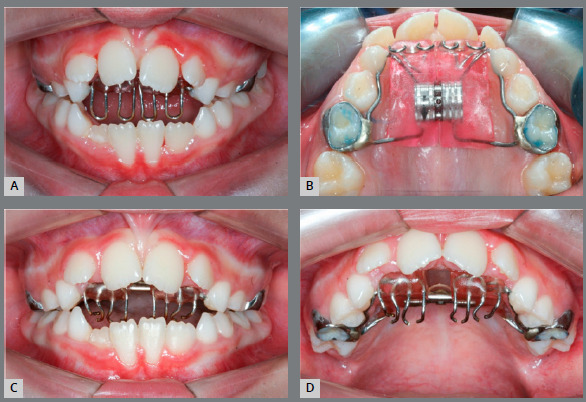
Haas appliance with grid installed, without activation.

The objectives of the first phase of this orthodontic treatment were: to correct the transverse maxillary arch discrepancy, to eliminate the thumb-sucking habit, to control the vertical growth tendency, and to close the anterior open bite, minimizing the complexity of the second phase of treatment, when fixed orthodontic appliances would be placed to achieve ideal esthetics and proper function. Treatment started with a modified Haas rapid palatal expander (RPE), that presented bands on the deciduous maxillary second molars and a crib inserted to the anterior portion of the acrylic pads (Figs 4A and 4B). 

Activation protocol was ¼ of a turn per day during three weeks, when the lingual cusps of the maxillary molars were touching the buccal cusps of the mandibular molars, thus the parents were oriented to stop activation. No device, brass wire or ligature, neither acrylic or composite resins were placed to lock the RPE screw. Two months later, the anterior open bite had decreased and the vertical loops of the crib were cut with a #1557 carbide bur, and it was transformed into a spur to further stimulate the elimination of the thumb and tongue thrusting habit (Figs 4C and 4D). 

**Figure 5:**
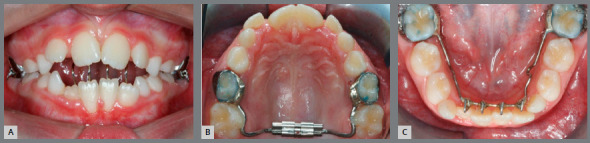
Anterior open bite six months after treatment onset (**A**). Mini-Hyrax used for second RME (**B**). Lingual arch with spurs (**C**).

**Figure 6:**
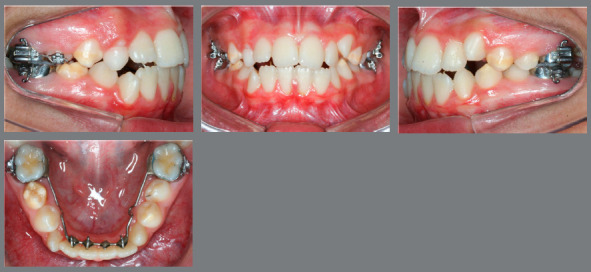
End of intervention with Mini-Hyrax appliance and spur.

After a 6-month RPE retention with the modified Haas appliance, there was a significant spontaneous decompensation of the mandibular molars’ lingual inclination. Therefore, a second maxillary expansion was performed with a Mini-Hyrax expander (1/4 turn/day for two weeks). The anterior open bite had been reduced in half, and a mandibular tongue spur was inserted to further assist in the open bite correction (Fig 5). After the 6-month second RPE retention, the Mini-Hyrax was removed, a fixed transpalatal arch was inserted to the maxillary permanent first molars, and a high-pull headgear was used at night for vertical growth control. At the end of the first phase, the transverse dimension deficiency and the anterior open bite had been corrected (Fig 6). The mandibular tongue spur was removed when all mandibular premolar had been fully erupted. 

**Figure 7:**
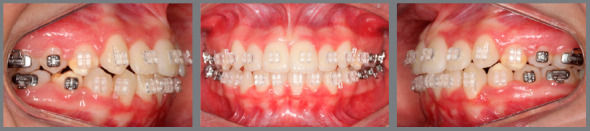
Intraoral photographs at alignment and leveling phase of dental arches.

The second phase of treatment started when all permanent second molars were fully erupted, and maxillary and mandibular fixed 0.022 x 0.028-in appliances were bonded on all mandibular and maxillary teeth (MBT Radiance brackets and Master Series Standard Edgewise tubes, American Orthodontics, Sheboygan, WI, USA). Leveling and alignment was achieved with 0.014-in NiTi, 0.018-in NiTi, 0.018-in SS wires and mild interproximal enamel reduction. Subsequently, 0.017 x 0.025-in and 0.019 x 0.025in TMA wires were used to improve torque control. At the beginning of the finishing phase, a panoramic radiograph was obtained to check the necessity to improve root parallelism, and progress photographs (Fig 7) and models were obtained to evaluate the need for any bracket repositioning, as previously reported.^15^ The BBO objective evaluating criteria (*https://bbo.org.br*) were used during the final stages of this orthodontic retreatment, to increase finishing efficiency and to optimize the achievement of excellent results. Fixed appliances were removed 15 months after the start of the second phase of treatment. Retention protocol consisted of a 0.036-in SS modified wraparound retainer on the maxillary arch and a 0.035-in Essix retainer on the mandibular arch. Patient was oriented to full-time wear of the retainers during the first six months post-debonding and night-time wear thereafter. 

#### TREATMENT RESULTS

**Figure 8:**
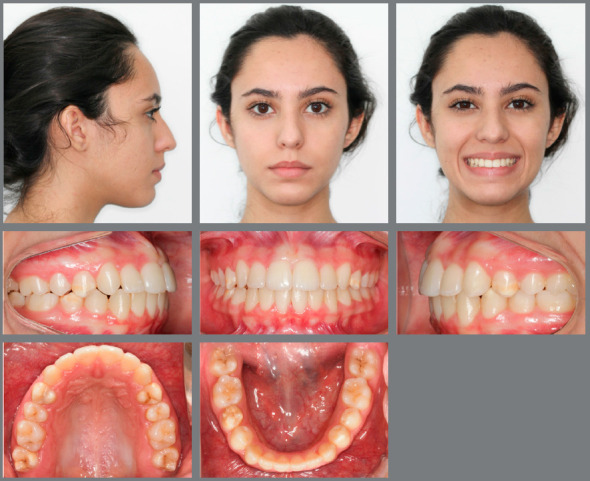
Final extraoral and intraoral photographs.

**Figure 9:**
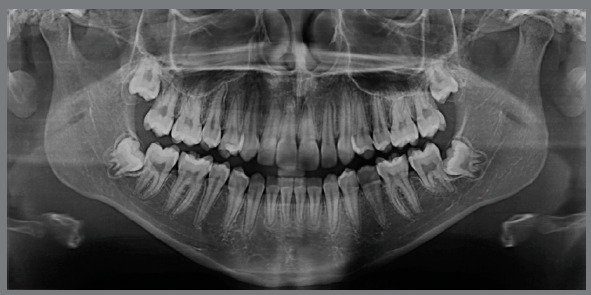
Final panoramic radiograph.

Treatment goals were achieved after the two phases of intervention. First of all, the thumb-sucking habit was controlled, and the major etiologic factor that caused her malocclusion was eliminated. In the facial aspect, passive lip sealing was obtained and there was a remarkable improvement on her smile esthetics, due to the proper correction of her smile arch and the improved proportions of the buccal corridors. The facial profile also improved and a better and well-defined chin-neck line was noted at the end of treatment (Fig 8). Intraoral post-treatment examination revealed that an ideal occlusion was achieved, with a Class I molar and canine relationship bilaterally, adequate overjet and overbite, coincident midlines and appropriate alignment and leveling of the marginal ridges (Fig 8). Final panoramic radiograph evaluation showed no signs of root resorption, adequate overall alveolar bone levels and appropriate root parallelism (Fig 9). 

**Figure 10:**
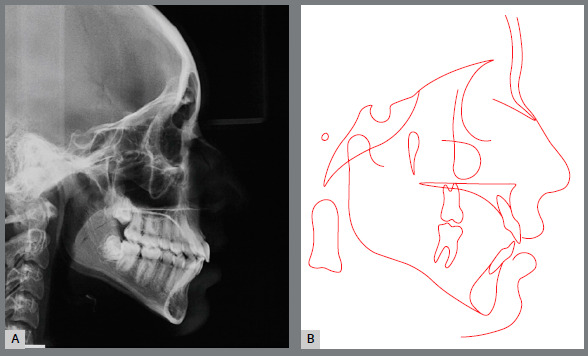
Final cephalometric profile radiograph (A) and cephalometric tracing (B).

**Figure 11:**
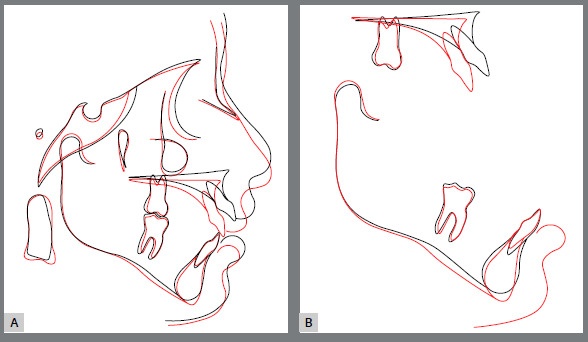
Initial (black) and post-treatment (red) total (**A**) and partial (**B**) superimpositions of cephalometric tracings.

**Figure 12:**
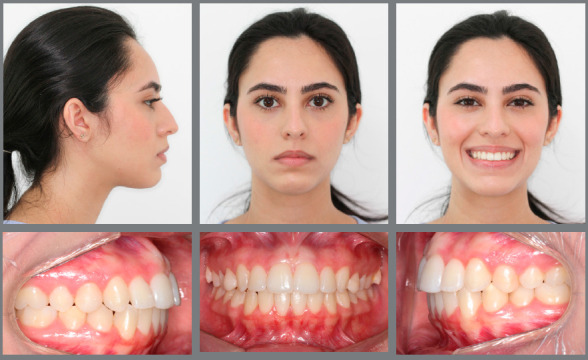
Four years after debonding, the patient’s occlusion remained stable.

Post-treatment cephalometric evaluation (Fig 10) confirmed the improvement of the anteroposterior discrepancy (ANB from 7º to 4º; Wits from +6 to 0 mm) and a good vertical dimension control, even without premolar extractions (SN.GoGn from 39º to 40º and FMA remained the same). Maxillary incisors presented a significant reduction on their labial inclination (1.NA from 32º to 27º), while the mandibular incisors were slightly flared (IMPA from 97º to 98º). Finally, no reduction on airway space was observed. Cephalometric superimpositions also confirmed the good vertical control during orthodontic mechanics, with the overall superimposition showing an improvement on maxillary spatial position, maxillary partial superimpositions confirming a better position of the incisors and the partial mandibular superimposition showing the maintenance of the incisors spatial position (Fig 11). Four years after treatment and two years without wearing any retainers, according to the patient, the results remained stable (Fig 12).

### CASE 2

#### DIAGNOSIS

A 21-year-old female sought orthodontic retreatment because she was very unhappy with her facial esthetics. She mentioned during her initial consultation how her excessive gummy smile and retrusive chin had been negatively impacting her self-esteem, her social relationships and her quality of life. The patient also stated that she didn’t like the esthetics of her nose, and the spaces between her anterior teeth compromised her smile and masticatory function. She explained that her previous orthodontic treatment took place was she was 12 years old and she wore fixed orthodontic appliances for approximately 3 years. 

**Figure 13:**
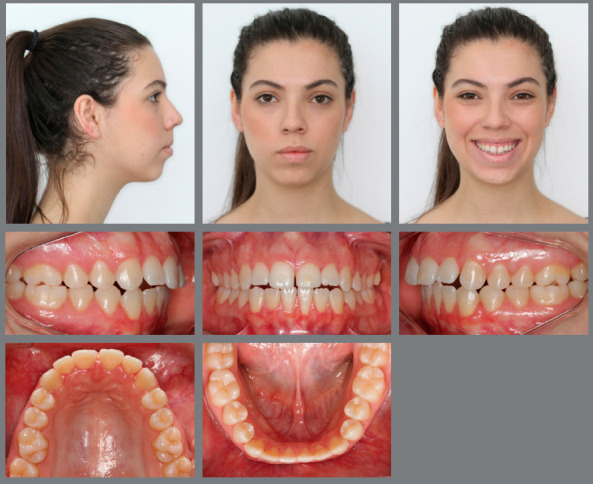
Initial extraoral and intraoral photographs.

Facial analysis revealed a severely convex profile, an excessive nasolabial angle, incompetent lips, a deep labiomental sulcus and a retruded chin. Frontal evaluation showed a symmetric face, increased facial height, with a long lower facial third and an excessive gingival display at smile (Fig 13). 

Intraoral evaluation showed a Class II, division 1 malocclusion, canines also in a Class II relationship, reduced anterior overbite and an increased overjet. The patient presented good oral hygiene, but a thin gingival phenotype in the mandibular incisors’ region (Fig 13).

**Figure 14:**
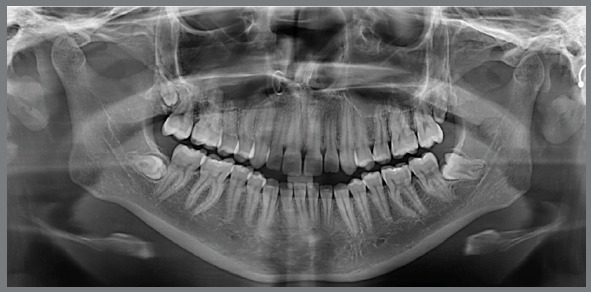
Initial panoramic radiograph.

**Figure 15:**
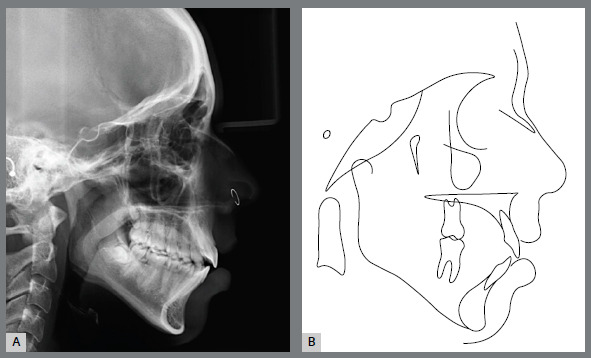
Initial lateral cephalometric radiograph (A) and cephalometric tracing (B).

Pre-retreatment panoramic radiograph showed that all permanent teeth were present. However, all third molars were impacted in an unfavorable position and without enough space for their eruption (Fig 14). Cephalometric analysis confirmed the facial diagnosis of a skeletal Class II (ANB = 8º; Wits = +10 mm), with a properly positioned maxilla in the sagittal plane (SNA = 82º) and a retruded mandible (SNB = 74º). The increased vertical dimension of her face was also confirmed (SN.GoGn = 41º, FMA = 32º). Maxillary incisors presented axial inclination within normal limits (1.NA = 22º), but were retroclined (1-NA = 1 mm). Finally, the mandibular incisor were both proclined (IMPA = 101º; 1.NB = 36º) (Fig 15 and Table 2).

**Table 2:** Cephalometric values at start (A) and at the end (B) of treatment.

	MEASURES		Normal	A	B	A/B
Skeletal pattern	SNA	(Steiner)	82°	82°	81°	1
	SNB	(Steiner)	80°	74°	77°	3
	ANB	(Steiner)	2°	8°	4°	4
	Wits	(Jacobson)	♀ 0 ± 2 mm ♂ 1 ± 2 mm	10 mm	1 mm	9
	Angle of convexity	(Downs)	0°	15°	9°	6
	Facial Angle	(Downs)	87°	81°	84°	3
	SN.GoGn	(Steiner)	32°	41°	34°	7
	FMA	(Tweed)	25°	32°	28°	4
Dental pattern	IMPA	(Tweed)	90°	101°	99°	2
	1.NA (degrees)	(Steiner)	22°	22°	22°	0
	1-NA (mm)	(Steiner)	4 mm	1 mm	4 mm	3
	1.NB (degrees)	(Steiner)	25°	36°	30°	6
	1-NB (mm)	(Steiner)	4 mm	10 mm	9°	1
	- Interincisal angle	(Downs)	130°	110°	112°	2
Profile	Upper lip - S-line	(Steiner)	0 mm	1 mm	-1 mm	2
	Lower lip - S-line	(Steiner)	0 mm	4 mm	- 0.5 mm	4.5

#### TREATMENT PLAN AND MECHANICS USED

The patient was very assertive during her initial consultation, and she understood the need and the potential benefits of an orthodontic retreatment. However, she was a little frustrated to face that reality and thus she made it clear that she wanted the shortest treatment possible, with the best possible results. Treatment objectives included: decrease the vertical dimension of her face, reduce her gummy smile, improve her facial profile and lip posture, achieve adequate Class I molar and canine relationships, and appropriate overjet and overbite. Consequently, her treatment planning included fixed orthodontic appliances, orthognathic surgery for maxillary impaction, mandibular advancement, counterclockwise rotation of the occlusal plane and a genioplasty. Furthermore, the multidisciplinary team involved in her treatment suggested the performance of a rhinoplasty at the same surgical intervention as the orthognathic surgery, what would eliminate the need for a second surgical procedure some months later.

**Figure 16:**
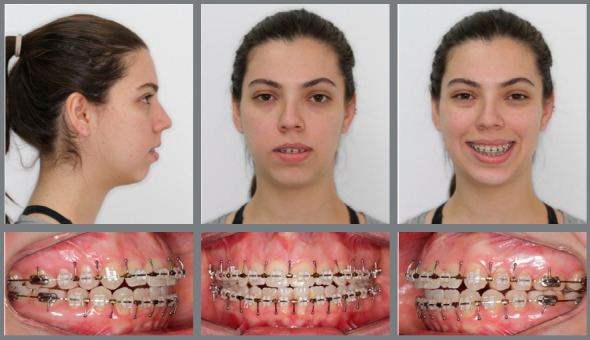
Presurgical orthodontic retreatment lasted 9 months.

Orthodontic retreatment was initiated with the bonding of 0.022 x 0.028-in brackets in both arches (MBT Radiance brackets and Master Series Standard Edgewise tubes, American Orthodontics, Sheboygan, WI, USA). Leveling and alignment was achieved with 0.018-in NiTi and 0.018-in SS. During the initial stages of the presurgical orthodontic phase, the patient also extracted all impacted third molars. Subsequently, 0.018 x 0.025-in TMA wires were used to improve torque control, and a 0.018 x 0.025-in SS wire was inserted two months prior to the orthognathic surgery. After the multidisciplinary team evaluated the surgical simulation with presurgical study models and were satisfied with the orthodontic preparation, surgical hooks were welded to the 0.018 x 0.025-in SS wire one week before surgery (Fig 16). Presurgical orthodontic retreatment lasted nine months. 

Orthognathic surgery and rhinoplasty took place as planned. The patient was seen by the orthodontist 1-, 2- and 4-weeks post-surgery. During the first two appointments, intermaxillary elastics were kept in the same position as determined by the oralmaxillofacial surgeon. However, one month post-surgery, both surgical wires were removed and 0.018-in SS wires were inserted, and intermaxillary elastics were used as needed to initiate the correction of the residual malocclusion. 

Four months after surgery, the finishing stages of the orthodontic retreatment started with the evaluation of a panoramic radiograph and finishing study models.^15^ Once again, the BBO objective evaluating (*https://bbo.org.br*) criteria was used to assist in achieving ideal finishing, and bracket repositioning was performed as needed. Post-surgical orthodontics lasted another nine months and the fixed appliances were removed after a total retreatment time of 18 months. Retention protocol consisted of a 0.035-in Essix retainer on the maxillary arch and a 0.018-in SS bonded to all mandibular six anterior teeth. Patient was oriented to full-time wear the Essix retainer during the first six months post-debonding, and night thereafter. 

#### TREATMENT RESULTS

**Figure 17:**
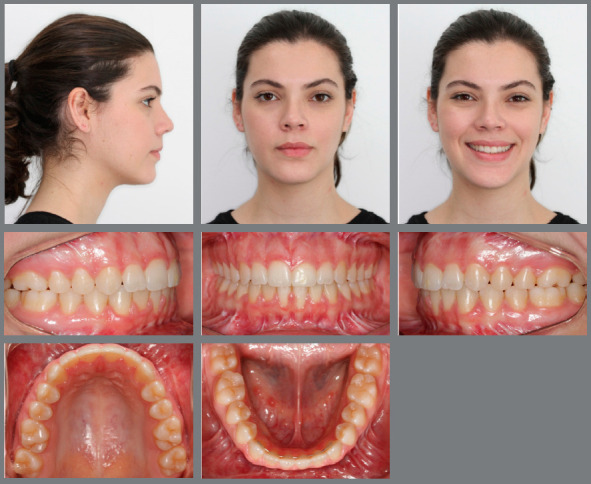
Final extraoral and intraoral photographs.

Treatment goals were achieved. Post-retreatment facial evaluation showed a significant improvement on the overall facial harmony. Vertical discrepancies were corrected, passive lip sealing was present, smile esthetics significantly improved, as seen with the amount of gingival display within normal limits. Profile analysis revealed a remarkable improvement, as seen with the Class I facial profile, better lip posture, equilibrated facial thirds and harmonic nose. Post-debonding intraoral analysis showed Class I molar and canine relationships, well-coordinated dental arches, adequate overbite and overjet (Fig 17). 

**Figure 18:**
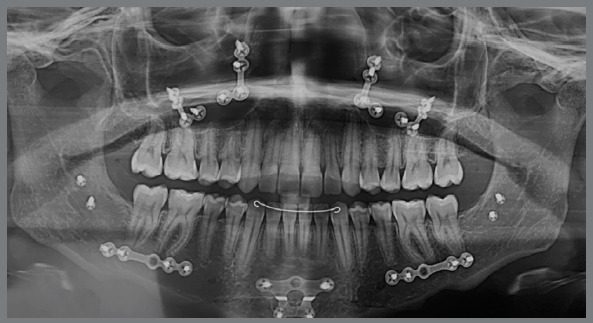
Final panoramic radiograph after treatment.

**Figure 19:**
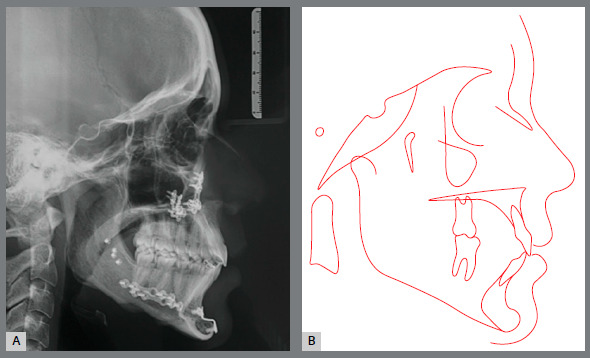
Final cephalometric profile radiograph (A) and cephalometric tracing (B).

**Figure 20:**
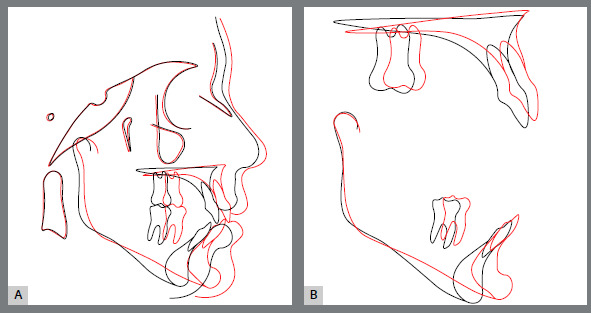
Initial (black) and post-treatment (red) total (**A**) and partial (**B**) superimpositions of cephalometric tracings.

**Figure 21:**
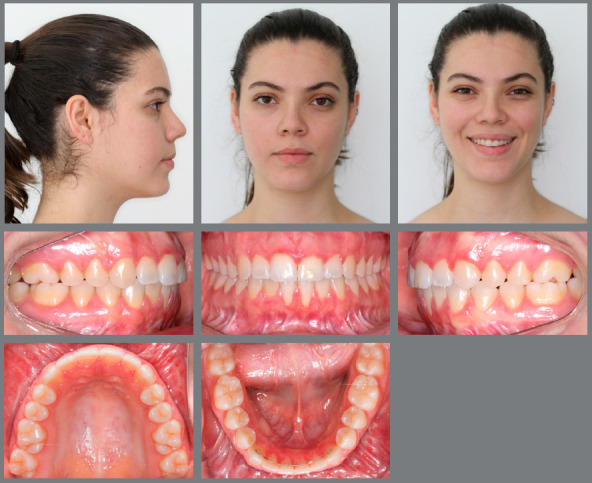
Four years after debonding, the patient’s occlusion remained stable.

Panoramic radiograph evaluation revealed adequate root parallelism, maintenance of normal alveolar bone levels no signs of root resorption or any sequelae from the surgical procedures (Fig 18). Cephalometric evaluation confirmed the improvement on both sagittal (ANB from 8º to 4º; Wits from 10 mm to 1 mm) and vertical planes (SN.GoGn from 41º to 34º; FMA from 32º to 28º). The spatial position and axial inclination of the maxillary incisors were maintained, and the labial inclination of the mandibular incisors was reduced (IMPA from 101º to 99º) (Fig 19, Table 2) The superposition of cephalometric tracings showed the results obtained after orthognathic surgery, with an improvement in the position of the maxilla after impaction and consequent counterclockwise rotation of the mandible after advancement, what significantly improved the patient’s facial pattern. The partial superimpostion of the maxillary tracings revealed an improvement in the position of the maxilla and consequent position of the incisors, in addition to the intrusion and mesial movement of molars. For the mandible, partial superimposition showed the extrusion and mesial movement of the molars, and the incisors remained in their position in relation to the bone base (Fig 20). Four years after orthodontic retreatment, the results remain stable and the patient continued to report high levels of satisfaction with the multidisciplinary treatment outcomes (Fig 21). 

## DISCUSSION

When we talk about health, we understand that it is a state of broad subjectivity, so when the subject extends to oral health, this is no different.^16^ ^,^ ^17^ Locker^18^ determined oral health as a condition that contributes to physical, psychological and social well-being. Therefore, the individuals would be able to eat, communicate, relate to their peers and consequently, exercise their roles in society without feeling uncomfortable nor embarrassed.^18^

Oral health-related quality of life (OHRQoL) has been assessed as the evaluation of the impact of oral conditions on individuals’ quality of life and well-being.^19^ OHRQoL is a multidimensional concept, which allows researchers and practitioners to understand the effects of oral outcomes on people’s life dimensions, such as symptoms, functioning, emotional and social well-beings.^20^ The increased demand for orthodontic treatment with fixed appliances in the general population has been reported in recent years.^3^ ^,^ ^19^ ^,^ ^21^ This interest has been justified, in particular, by the population’s growing access to dental services. Furthermore, an increasing number of individuals have made the association between poor oral health and psychosocial problems, which characterizes this population’s understanding of their oral problems.^2^ ^,^ ^4^

However, one of the great debates in adult Orthodontics regards the challenges associated with long-term post-treatment stability. A large amount of evidence has demonstrated that even when the orthodontist is able to achieve good occlusion, relapse is a matter.^5^ ^,^ ^11^ ^,^ ^22^ The scenario becomes worse if iatrogenic issues take place and the orthodontist is inattentive to adequate canine and lateral guidance, as well as appropriate alignment and intercuspation during orthodontic finishing.^5^ ^,^ ^23^

The literature seems to recognize that individuals seeking orthodontic retreatment present themselves disappointed and demotivated during their appointments with the orthodontist.^12^ ^,^ ^13^ ^,^ ^24^ However, the well-being of these individuals and the impact of orthodontic retreatment on OHRQoL have been not been fully discussed in the literature.^12^ The two cases reported in this paper represent good examples of how orthodontic retreatments that achieve well-planed goals may significantly improve the patients’ self-esteem. We can see the positive effect of orthodontic therapy on both the functional and aesthetic aspects of both patients, and this in fact results in a positive impact on the quality of life after a second orthodontic intervention. This is because it is known that oral health problems are directly related to negative self-perception of appearance, leading to deterioration of emotional and social behavior.^25^ ^,^ ^26^

Despite the self-reported perception and complaint of these individuals regarding their dental problems, the willingness to undergo orthodontic treatment again may lead to insecurity and uncertainties. Anxiety levels of individuals who are about to begin orthodontic treatment are high and probably negatively influence health-related quality of life.^27^ ^,^ ^28^ On the other hand, the encouragement and the positive reinforcement that comes from the orthodontist may be helpful for the individual in overcoming his/her negative perception and, ultimately, decides to undergo orthodontic retreatment.^23^ ^,^ ^29^

## CONCLUSION

The population’s growing search for orthodontic treatment is of great importance and interest for orthodontists, however we must be aware of the patient’s interests and especially of our capacity to perform treatments that bring positive results to their demands. Listening to the patient and knowing the best time for the intervention is certainly the best path for the success of orthodontic treatment, thus avoiding the need for new future interventions.
